# BHLHE41 inhibits bladder cancer progression via regulation of PYCR1 stability and thus inactivating PI3K/AKT signaling pathway

**DOI:** 10.1186/s40001-024-01889-2

**Published:** 2024-05-29

**Authors:** Shuai Xiao, Junjie Chen, Yongbao Wei, Wei Song

**Affiliations:** 1 Department of Urology, Hunan Provincial People’s Hospital, The First Affiliated Hospital of Hunan Normal University, Changsha, 410011 China; 2 Department of Urology, Fujian Provincial Hospital, Shengli Clinical Medical College of Fujian Medical University, Fuzhou, 350001 China

**Keywords:** BHLHE41, Bladder cancer, PYCR1, PI3K/AKT signaling pathway, Ubiquitination

## Abstract

**Background:**

The basic helix-loop-helix family member e41 (BHLHE41) is frequently dysregulated in tumors and plays a crucial role in malignant progression of various cancers. Nevertheless, its specific function and underlying mechanism in bladder cancer (BCa) remain largely unexplored.

**Methods:**

The expression levels of BHLHE41 in BCa tissues and cells were examined by qRT-PCR and western blot assays. BCa cells stably knocking down or overexpressing BHLHE41 were constructed through lentivirus infection. The changes of cell proliferation, cell cycle distribution, migration, and invasion were detected by CCK-8, flow cytometry, wound healing, transwell invasion assays, respectively. The expression levels of related proteins were detected by western blot assay. The interaction between BHLHE41 and PYCR1 was explored by co-immunoprecipitation analysis.

**Results:**

In this study, we found that BHLHE41 was lowly expressed in bladder cancer tissues and cell lines, and lower expression of BHLHE41 was associated with poor overall survival in bladder cancer patients. Functionally, by manipulating the expression of BHLHE41, we demonstrated that overexpression of BHLHE41 significantly retarded cell proliferation, migration, invasion, and induced cell cycle arrest in bladder cancer through various in *vitro* and in *vivo* experiments, while silence of BHLHE41 caused the opposite effect. Mechanistically, we showed that BHLHE41 directly interacted with PYCR1, decreased its stability and resulted in the ubiquitination and degradation of PYCR1, thus inactivating PI3K/AKT signaling pathway. Rescue experiments showed that the effects induced by BHLHE41 overexpression could be attenuated by further upregulating PYCR1.

**Conclusion:**

BHLHE41 might be a useful prognostic biomarker and a tumor suppressor in bladder cancer. The BHLHE41/PYCR1/PI3K/AKT axis might be a potential therapeutic target for bladder cancer intervention.

## Introduction

Bladder cancer (BCa) is a prevalent and deadly kind of cancer that affects the genitourinary system. According to statistics, there were about 573,000 new cases and 213,000 deaths recorded globally in 2020, indicating a significant societal impact [1]. The development of BCa was shown to be influenced by environmental risk factors such as cigarette smoking and prolonged exposure to paint components [2]. The independent risk factors for BCa were age and male sex, while protective factors were cessation of smoking and presence of cardiovascular disease (CVD) [3]. Advanced age at diagnosis appears to be associated with an increased risk of recurrence and progression [4]. Based on the extent of the tumor cells infiltrating into the bladder muscle wall, approximately 25% of BCa cases were initially diagnosed as muscle-invasive bladder cancer (MIBC), which had a property of rapid growth and metastasis [5, 6]. The remaining cases were non-muscle-invasive bladder cancer (NMIBC), 15% of which would eventually develop into MIBC [7, 8]. The existing conventional approaches, including the transurethral resection of bladder tumor (TURB) followed by intravesical immunotherapy with the bacillus of Calmette-Guerin (BCG) or chemotherapy, depending on the grade of bladder tumor, have consistently advanced over the last several decades and have shown to be efficacious for patients in the early stages of the disease [9–11]. Regrettably, the outlook was quite unfavorable for patients with advanced BCa as a result of distant metastases, even with the implementation of systematic therapy [12–14]. Genomic alterations have been increasingly recognized as crucial factors in the advancement and spread of bladder cancer. Therefore, there is a pressing need to gain a more profound comprehension of the fundamental molecular pathways implicated in BCa, since this might potentially pave the way for the creation of more effective anticancer therapies.

Basic helix-loop-helix family member e41 (*BHLHE41*), also called SHARP1, DEC2 and BHLHB3, is found on human chromosome 12p12.1. It encodes a 482 amino acid helix-loop-helix transcription factor. BHLHE41 has previously been shown to exert an important role in maintaining circadian rhythm and regulating cell differentiation and death via transcription repression or other mechanisms [15–17]. Dysregulation of BHLHE41 has been identified in a variety of cancers, which contributes to the formation and progression of tumors and may be used as a prognostic marker [18]. In breast cancer, *Zhang *et al. [19] found that BHLHE41 inhibited tumor invasion by inactivating the MAPK/JNK signaling pathway, which was accompanied by downregulation of SNAI1, SNAI2, VIM, and CDH2 and overexpression of CLDN1, CLDN4, and CDH1. In gastric cancer (GC), BHLHE41 expression was downregulated in GC tissues, and lower expression was associated with higher TNM stage and worse overall survival of GC patients. Overexpression of BHLHE41 inhibits GC cell development in vitro and in vivo by inhibiting the ERK/NF-κB/EMT axis [20]. Furthermore, BHLHE41 was shown to have an oncogenic or suppressive function in lung cancer, clear cell renal cell carcinoma, and endometrial cancer [21–23]. However, the relevance and underlying mechanism of BHLHE41 in bladder cancer have not been studied.

In this work, our team discovered for the first time that BHLHE41 was downregulated in bladder cancer tissues and cells, and had a favorable correlation with a good prognosis in bladder cancer patients. We discovered that BHLHE41 is a tumor suppressor in bladder cancer and conducted in *vitro* and in *vivo* investigations to show its influence on cell proliferation, migration, invasion, and cell cycle progression. Mechanistically, we established that PYCR1 is a direct substrate of BHLHE41 and mediates BHLHE41's influence on the PI3K/AKT signaling pathway activity. Overall, our findings identified the BHLHE41-PYCR1-PI3K-AKT axis as a regulator of bladder cancer growth and gave new insights into possible therapeutic targets for bladder cancer.

## Material and methods

### Sample collection

Paired BCa tissues and corresponding normal tissues were collected in the Department of Urology, Hunan Provincial People’s Hospital between August 2021 to July 2023, with the approval by the Research Ethics Committee of Hunan Provincial People’s Hospital. The written informed consent was obtained from each patient.

### Cell lines and cell culture

The BCa cell lines (T24, 5637, and UM-UC-3) and the human embryonic kidney cell line HEK-293 T were obtained from the cell bank of the Chinese Academy of Sciences (Shanghai, China). The normal urothelial cell line SV-HUC-1 was obtained from the Procell Life Science and Technology (Wuhan, China). These cell lines were separately cultured in McCoy's 5a (T24), RPMI 1640 (5637 and SV-HUC-1), and DMEM (UM-UC-3 and HEK-293 T) in an incubator at 37 °C, in the presence of 5% CO_2_. All the culture media were supplemented with 10% fetal bovine serum (FBS, Gibco, USA) and 1% antibiotics (penicillin/streptomycin) (Servicebio, China).

### Construction of stably cell lines

Stable knockdown or overexpression of BHLHE41 in T24 and 5637 cells were performed through lentivirus infection. Briefly, the shBHLHE41 and LV- BHLHE41 lentivirus and their corresponding negative control lentivirus (shNC and LV-Control) were bought from OBiO Technology (Shanghai, China), and were used to infect T24 and 5637 cells according to manufacturer’s instructions. Twenty-four hours after infection, the supernatants were removed and fresh medium containing puromycin (3 mg/mL) were added for further selection for 5 days. The stably infected cells were confirmed via qRT-PCR and western blot assays.

### Plasmid transfection

Full-length BHLHE41 and PYCR1 were cloned into the pcDNA3.1-3x-FlAG and pcDNA3.1-HA vector, respectively, to generate the overexpression plasmids. For plasmid transfection, a total of 5 μg plasmid and 10 μl Lipofectamine^®^3000 were diluted in 200 μl Opti-MEM culture medium and the mixtures were further used to transduce cells. Twelve hours after transfection, the supernatants were replaced with medium containing 10% FBS and cultured for 36 h before being used for further experiments.

### CCK-8 assay

Cell proliferation ability was evaluated by CCK-8 assay. Briefly, each group of cells were seeded in 96-well plates at a density of 2 × 10^3^ per well. At 0, 24, 48, and 72 h, cells in each well were incubated with 10 μl CCK-8 solution reagent (Dojindo, Japan) for 2 h in the incubator. After that, the absorbance values at the wavelength of 450 nm were measured using a microplate reader (Bio-rad, USA).

### Cell cycle assay

The cell cycle distribution was determined using propidium iodide (PI) reagent (Sigma, USA) following the manufacturer’s instructions. Briefly, stably infected cells were collected, and were then fixed in 75% ethanol at 4 °C overnight. In the next day, cells were incubated with PI reagent (50 μg/mL) for 30 min in the dark after being treated with RNase (1 mg/mL) for 30 min. Finally, a FACS flow cytometer (Becton, Dickson and Company, USA) was used to examine the cell cycle distribution of each sample.

### Wound healing assay

Cell migration ability was assessed by wound healing assay as previously described [24]. In brief, each group of cells were cultured in 6-well plates until reaching 100% density. Then, the supernatants were removed and the cells were scratched vertically with a sterile 10-µl pipette tip before being washed with PBS twice. Subsequently, the serum free medium was added into the plate. The cells were observed and photographed under an inverted microscope (Olympus, Japan) at 0 and 36 h.

### Transwell invasion assay

To determine cell invasion ability under different conditions, each group of cells were suspended in serum-free medium at a density of 5 × 10^4^/ml. Then, 200 µl cell suspension were added into the Matrigel-coated transwell chambers (Corning, USA) in 24-well plates. Afterwards, 600 µl medium containing 20% FBS were supplemented into the outside of the chamber. After being cultured for 36 h in the incubator, the invaded cells in the lower chamber were fixed with 4% paraformaldehyde and cells in the upper chamber were removed using the cotton swab. Then, cells were stained with 0.1% crystal violet (Servicebio, China) for 20 min at room temperature, followed by being washed with PBS twice. The invaded cells were observed and photographed using a light microscope (Olympus, Japan). The number of invaded cells were determined by ImageJ software.

### RNA isolation and qRT-PCR

Total RNA in tissues or cells were isolated with TRIzol reagent (Invitrogen; Thermo Fisher Scientific) according to the manufacturer’s protocol. After determining its concentration, a total of 1 µg RNA was reversely transcribed into cDNA using PrimeScript RT Master Mix (Takara, Japan). qRT-PCR was conducted using the iQTM SYBR^®^ Green Supermix (Bio-Rad, USA) with the CFX96TM Real-time PCR Detection System (Bio-Rad, USA). Each reaction of qRT-PCR was performed with 10 µl of iQTM SYBR^®^ Green Supermix, 2 µl of cDNA, 2 µl of primer, and 6 µl of nuclease-free H_2_O. The expression level of *GAPDH* was regarded as endogenous control and the relative gene expression was calculated by 2^−ΔΔCt^ method. The corresponding primer sequences were list as follow: BHLHE41, forward primer, 3′-AGCTTTAACCGCCTTAACCG-5′, reverse primer, 3′-GGTTGATCAGCTGGACACAC-5′; PYCR1, forward primer, 3′-CGTCACCATCAGCTCCATTG-5′, reverse primer, 3′-CGGCATCAATCAGGTCCTCT-5′; GAPDH, forward primer, 3′-CCATCTTCCAGGAGCGAGAT-5′, reverse primer, 3′-TGCTGATGATCTTGAGGCTG-5′.

### Protein isolation and western blot

Briefly, tissues and cells were lysed in cold RIPA buffer supplemented with 1% protease inhibitor and phosphatase inhibitor (MCE, USA) for half-hour in ice. After determining the protein concentration using Bradford protein assay kit (Beyotime, China), a total of 20 µg proteins were utilized for 10% SDS-PAGE. Then, the proteins were electro-transferred onto PVDF membrane (Millipore, German). Afterwards, the membranes were incubated with 5% nonfat milk for 1 h at room temperature for blocking. After washing with TBST three times, the primary antibodies were added into the membranes and incubated at 4 °C overnight. The next day, the membranes were washed with TBST three times and incubated with the peroxidase-conjugated second antibodies at room temperature for 1 h. Finally, the immunoreactive protein signal was visualized using a ECL kit (Beyotime, China) and photographed by the electromagnetic interference XRS imaging system (Bio-Rad, USA). The relative protein levels were determined using the ImageJ software.

### *Coimmunoprecipitation (Co-IP) assay and *In vivo* ubiquitination assay*

The interaction between BHLHE41 and PYCR1 were determined by Co-IP assay using the Pierce Co-Immunoprecipitation Kit (Termo Fisher, USA) according to the manufacturer’s instruction. Briefly, cells were collected and lysed in cold IP-lysis buffer for 20 min in ice. Then, the cell lysis solution was centrifugated at 12000 rpm and 4 °C for 10 min and the cell supernatant was retained. Protein G-agarose beads pre-incubated with indicated antibodies were added into the cell supernatant and further incubated overnight at 4 °C with gentle shaking. The next day, the immunoprecipitated proteins were washed with IP-lysis buffer and diluted in 1 × SDS buffer. After cooking at 95 °C for 10 min, the Co-IP proteins were subjected to western blot analysis. As for the in vivo ubiquitination assay, cells of each group were treated with MG132 (20 µM; MCE, USA) for 6 h before the Co-IP assay. PYCR1 specific antibody was utilized to immuno-precipitate proteins. Western blotting was performed using Anti-Ub (1:500, ABclonal) as the primary antibody.

### Bladder *cancer* xenograft animal model

Four-week-old BALB/C nude mice bought from the GemPharmatech biotechnology company (Nanjing, China) were reared in the pathogen-free animal center at our hospital. Equal number of T24 cells (stably transfected with LV-Ctrl or LV-BHLHE41 lentivirus) were resuspended in 200 PBS and were subcutaneously injected into the right flank of each mouse. Every week, the tumor volumes were determined using the following formula: Volume = 1/2 × (length) × (width)^2^. At the end of the animal experiments, mice were sacrificed by cervical dislocation. The xenografts were separated, weighted, and photographed before being fixed in 4% paraformaldehyde or stored in liquid nitrogen for subsequent analyses.

### Data mining and bioinformatic analysis

The TIMER (https://cistrome.shinyapps.io/timer/) and GEPIA (http://gepia.cancer-pku.cn/) databases were used to investigate the expression profile of BHLHE41 in various kinds of malignancies, as well as its prognostic implications in BLCA. We obtained a gene expression data matrix of BLCA samples from the TCGA database (https://portal.gdc.cancer.gov/) and divided it into BHLHE41^high^ and BHLHE41^low^ groups based on the median expression level of BHLHE41. The *edgeR* package in R was used to identify differentially expressed genes (DEGs) between BHLHE41^high^ and BHLHE41^low^ groups using the criterion of | log2(fold change) |> 1 and FDR < 0.05. The DAVIAD database (https://david.ncifcrf.gov/) was used to perform Gene Ontology (GO) and Kyoto Encyclopedia of Genes and Genomes (KEGG) enrichment analyses. The data was visualized using R packages including *pheatmap* and *ggplot2*.

### Statistical analysis

Data from at least three independent experiments were expressed as Mean ± standard deviation (SD). Statistical significance was evaluated by R 4.3.2 and GraphPad Prism 9.0 software. Student’s t test or one-way analysis of variance (ANOVA) were used to compare difference between two or multiple groups, respectively. We considered *P* < 0.05 as statistically significant.

## Results

### BHLHE41 was downregulated in bladder *cancer* and negatively correlated with clinical outcome

**Fig. 1:**
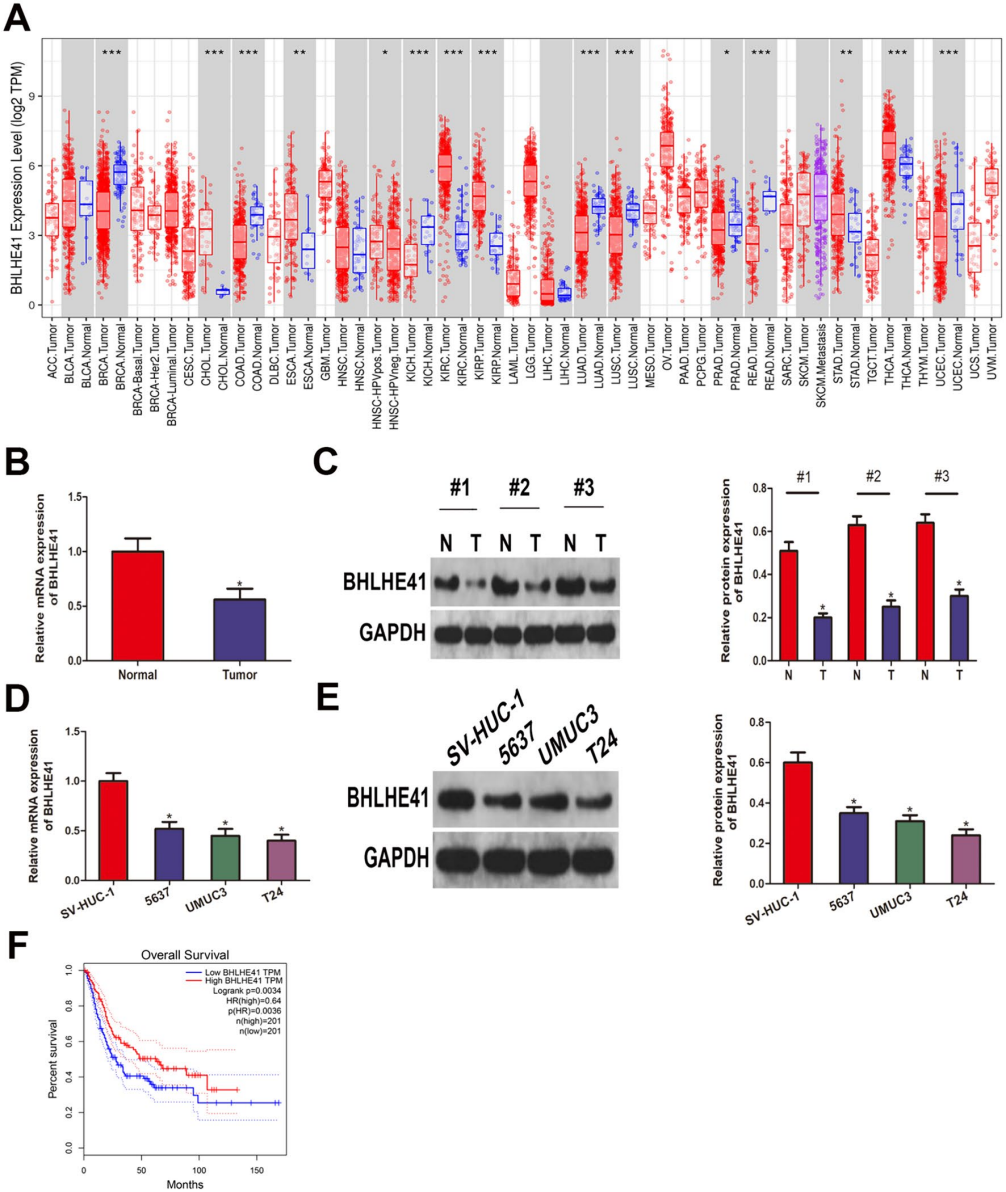
BHLHE41 was downregulated in bladder cancer and negatively correlated with clinical outcome. **A** BHLHE41 expression level in various types of tumors and corresponding normal tissues. **B** qRT-PCR was performed to evaluate BHLHE41 mRNA expression in BLCA tissues and paired normal tissues. **C** Western blot analysis of BHLHE41 protein levels in BLCA tissues and paired normal tissues, and quantitative analysis. **D** qRT-PCR analysis of BHLHE41 mRNA level in BLCA cell lines (T24, UMUC3, and 5637) and human bladder epithelial cells SV-HUC-1. **E** The comparison of BHLHE41 protein levels in cell lines including T24, UMUC3, 5637, and SV-HUC-1, and quantitative analysis. **F** Kaplan–Meier survival analysis of BHLHE41 in BLCA. *P < 0.05

First, we examined the expression profile of BHLHE41 in several kinds of malignancies using the TIMER database and discovered that BHLHE41 expression was commonly dysregulated in tumors. As shown in Fig. 1A, when compared to their equivalent normal tissues, BHLHE41 was abundantly expressed in CHOL, ESCA, KIRC, KIRP, STAD, and THCA, while it was downregulated in BLCA, BRCA, COAD, KICH, LUAD, LUSC, PRAD, READ, and UCEC. Consistent with the results in dataset, the mRNA and protein levels of BHLHE41 were lower in bladder cancer tissues than that in adjacent normal tissues, as validated by qRT-PCR, and western blot tests (Fig. 1B–C). Furthermore, we examined the expression of BHLHE41 in BCa cell lines (T24, UMUC3, and 5637) and human bladder epithelial cells SV-HUC-1, and discovered that BHLHE41 was downregulated in BCa cell lines (Fig. 1D–E). Finally, we stratified bladder cancer patients in TCGA dataset into BHLHE41^high^ and BHLHE41^low^ groups based on the median expression level of BHLHE41, and performed Kaplan–Meier survival analysis, and we found that patients with lower BHLHE41 expression had worse overall survival than those with higher BHLHE41 expression (Fig. 1F). Collectively, these findings indicated that BHLHE41 was downregulated in bladder cancer and negatively correlated with patients’ prognosis.

### BHLHE41 overexpression suppressed cell proliferation, migration, invasion, and induced cell cycle arrest in BCa cells

**Fig. 2:**
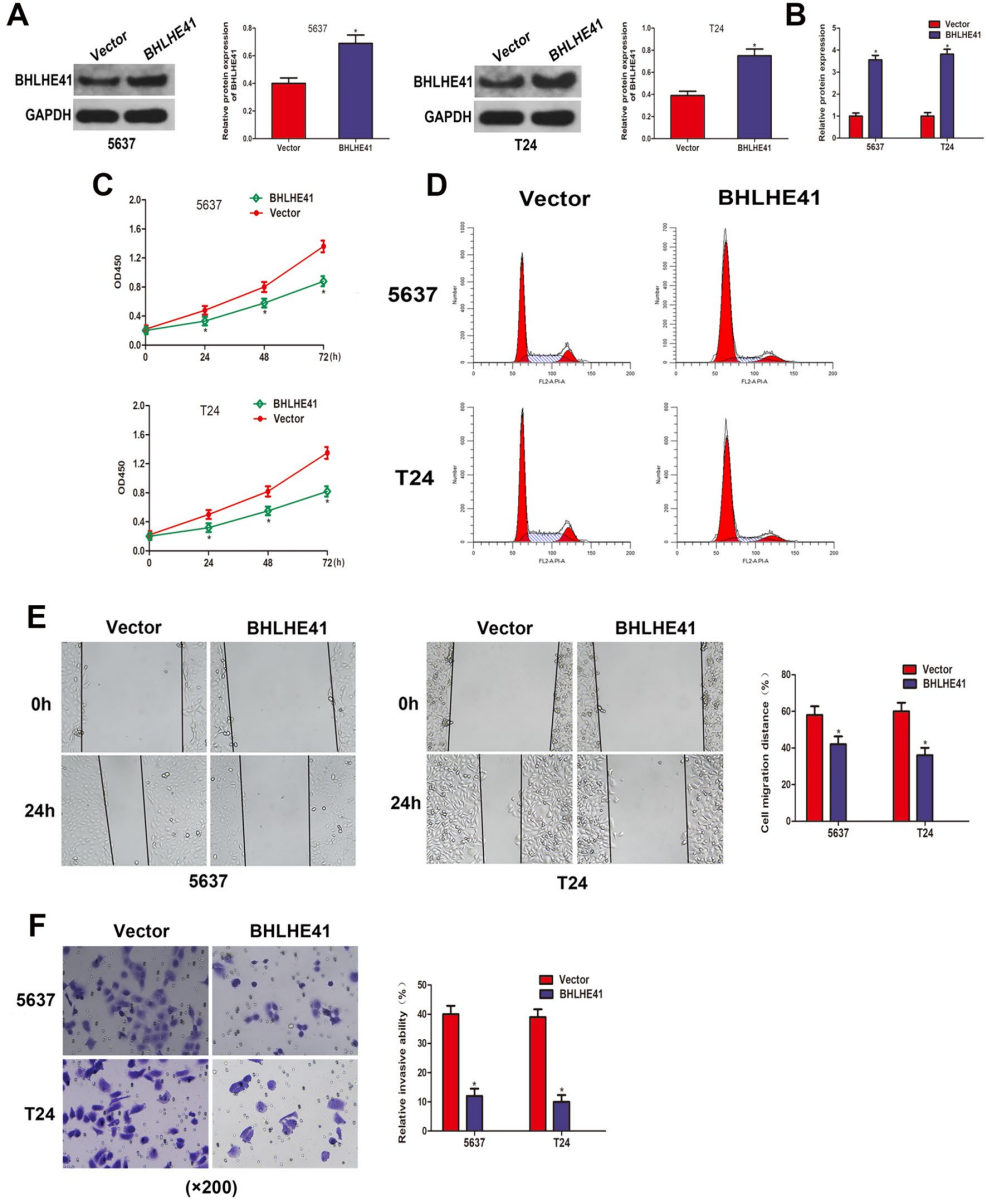
BHLHE41 overexpression suppressed cell proliferation, induced cell cycle arrest, and inhibited cell migration and invasion in BCa cells. **A** Western blot analysis of BHLHE41 protein level in 5637 and T24 cells stably infected with BHLHE41-overexpression or control lentivirus, and quantitative analysis. **B** qRT-PCR assay was employed to evaluate the overexpression efficiency in 5637 and T24 cells stably infected with BHLHE41-overexpression lentivirus. **C** CCK-8 assay assessed the proliferation abilities of 5637 and T24 cells after overexpressing BHLHE41. **D** The effect of BHLHE41 overexpression on cell cycle distribution of 5637 and T24 cells was detected by flow cytometry analysis. **E** Wound healing assay showed that the migration ability was impaired after overexpressing BHLHE41. The represent images and quantitative analysis. **F** Invasion ability of BCa cells infected with BHLHE41-overexpression or control lentivirus was determined by transwell invasion assay. *P < 0.05

To examine the functional roles of BHLHE41 in bladder cancer, we established the stable BHLHE41-overexpressing BCa cells through lentivirus infection, the efficiency of which was demonstrated via western blot and qRT-PCR assays (Fig. 2A–B). As shown in Fig. 2C, the OD values in LV-BHLHE41 group were significantly lower than those in LV-Ctrl group in the CCK-8 test, showing reduced cell proliferation capacity following overexpression of BHLHE41. To quantify the effect of BHLHE41 overexpression on cell cycle distribution in BCa cells, we performed flow cytometry analysis and discovered that overexpressing BHLHE41 increased percentage of cells in G0/G1, while decreasing the proportion of cells in S phase (Fig. 2D), implying that BHLHE41 overexpression induced cell cycle arrest at G0/G1 phase. Meanwhile, enforced expression of BHLHE41 notably inhibited the migration ability of T24 and 5637 cells, as implicated by decreased cell migration rate in the wound healing assay (Fig. 2E). Furthermore, the transwell invasion experiment demonstrated a reduction in the number of invaded cells in the LV-BHLHE41 group compared to LV-Ctrl group (Fig. 2F), indicating that the invasion capacity was significantly lowered when BHLHE41 was increased. Taken together, these results indicated that BHLHE41 suppressed cell proliferation, migration, invasion, and induced cell cycle arrest at G0/G1 in BCa cells.

### BHLHE41 deficiency promoted cell proliferation, migration, invasion, and accelerated cell cycle progression in BCa cells

**Fig. 3:**
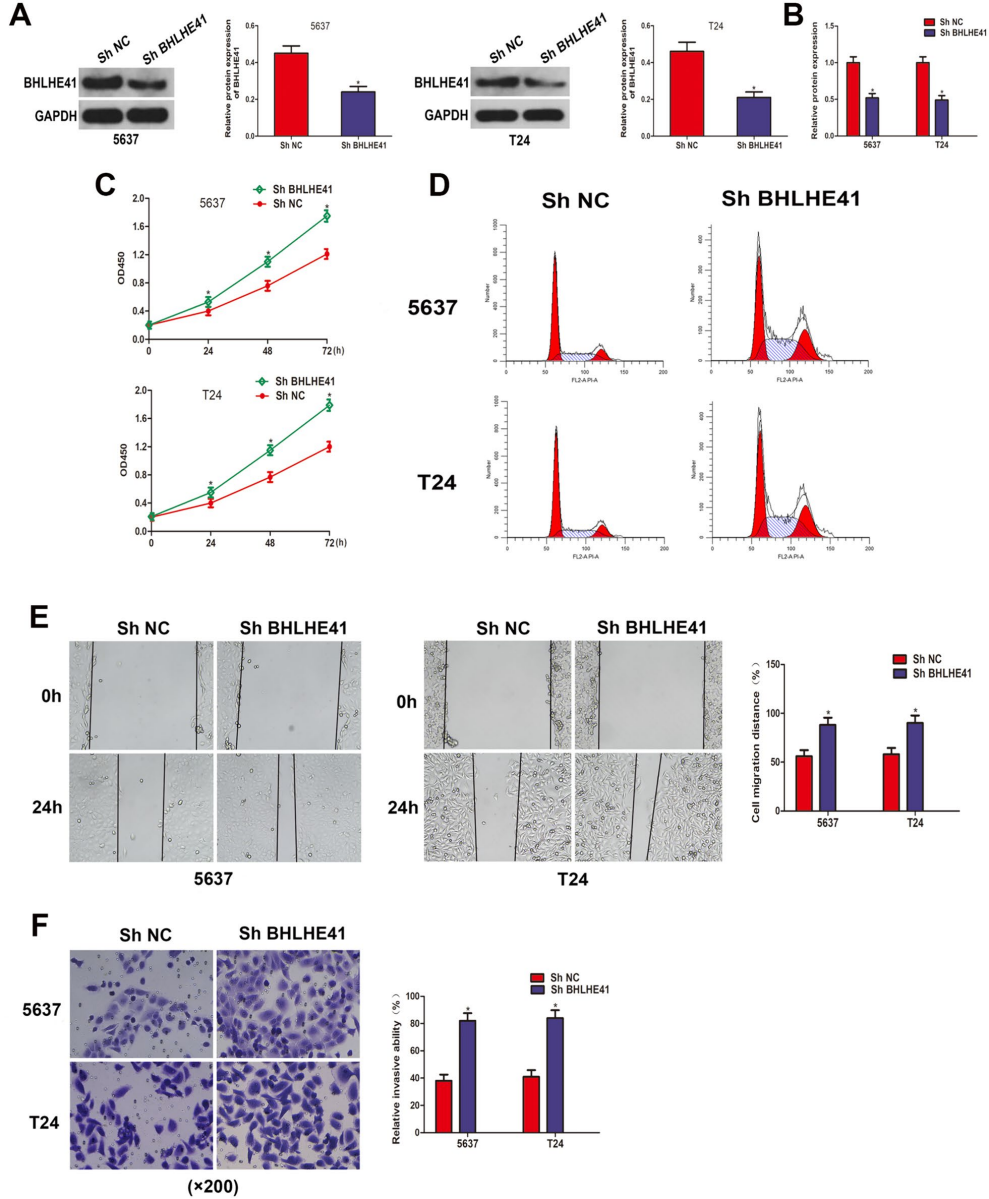
BHLHE41 deficiency promoted cell proliferation, accelerated cell cycle progression, and increased cell migration and invasion in BCa cells. **A** Western blot analysis of BHLHE41 protein level in 5637 and T24 cells stably infected with lentivirus containing specific shRNA targeting BHLHE41 (shBHLHE41) and control shRNA (shNC), and quantitative analysis. **B** qRT-PCR assay was employed to evaluate the knockdown efficiency in 5637 and T24 cells stably infected with LV-BHLHE41. **C** CCK-8 assay assessed the proliferation abilities of 5637 and T24 cells after knockdown of BHLHE41. **D** The effect of BHLHE41 knockdown on cell cycle distribution of 5637 and T24 cells was detected by flow cytometry analysis. **E** Wound healing assay showed that the migration ability was increased after knocking down BHLHE41. The represent images and quantitative analysis. **F** Invasion ability of BCa cells infected with lentivirus containing shBHLHE41 or shNC was determined by transwell invasion assay. *P < 0.05

To further determine the physiological function of BHLHE41 in BCa cells, BHLHE41 was stably knocking down in T24 and 5637 cells through lentivirus infection. Western blot and qRT-PCR assays confirmed the efficiency of the specific shRNA targeting BHLHE41 in BCa cells (Fig. 3A–B). The impact of BHLHE41 deficiency on cell proliferation was assessed via CCK-8 assay. As shown in Fig. 3C, higher OD values were observed after BHLHE41 abrogation, suggesting that BHLHE41-deficient BCa cells proliferated at a higher rate than the corresponding control cells. Furthermore, flow cytometry assay revealed that the proportion of cells in G2 phase was higher in shBHLHE41 group than that in shNeg group (Fig. 3D), suggesting accelerated cell cycle progression in BCa cells following deficiency of BHLHE41. Subsequently, we discovered that the migration rate in the wound healing experiment was greater in the shBHLHE41 group compared to the shNeg group (Fig. 3E), showing that BCa cells stably knocking down BHLHE41 displayed enhanced migratory ability. As expected, transwell assays demonstrated that when BHLHE41 was silenced, the invasion capability of BCa cells was significantly increased (Fig. 3F). Altogether, these results indicated that BHLHE41 deficiency promoted cell proliferation, migration, invasion, and accelerated cell cycle progression in BCa cells.

### BHLHE41 negatively regulated PI3K/AKT signaling pathway in BCa cells

**Fig. 4:**
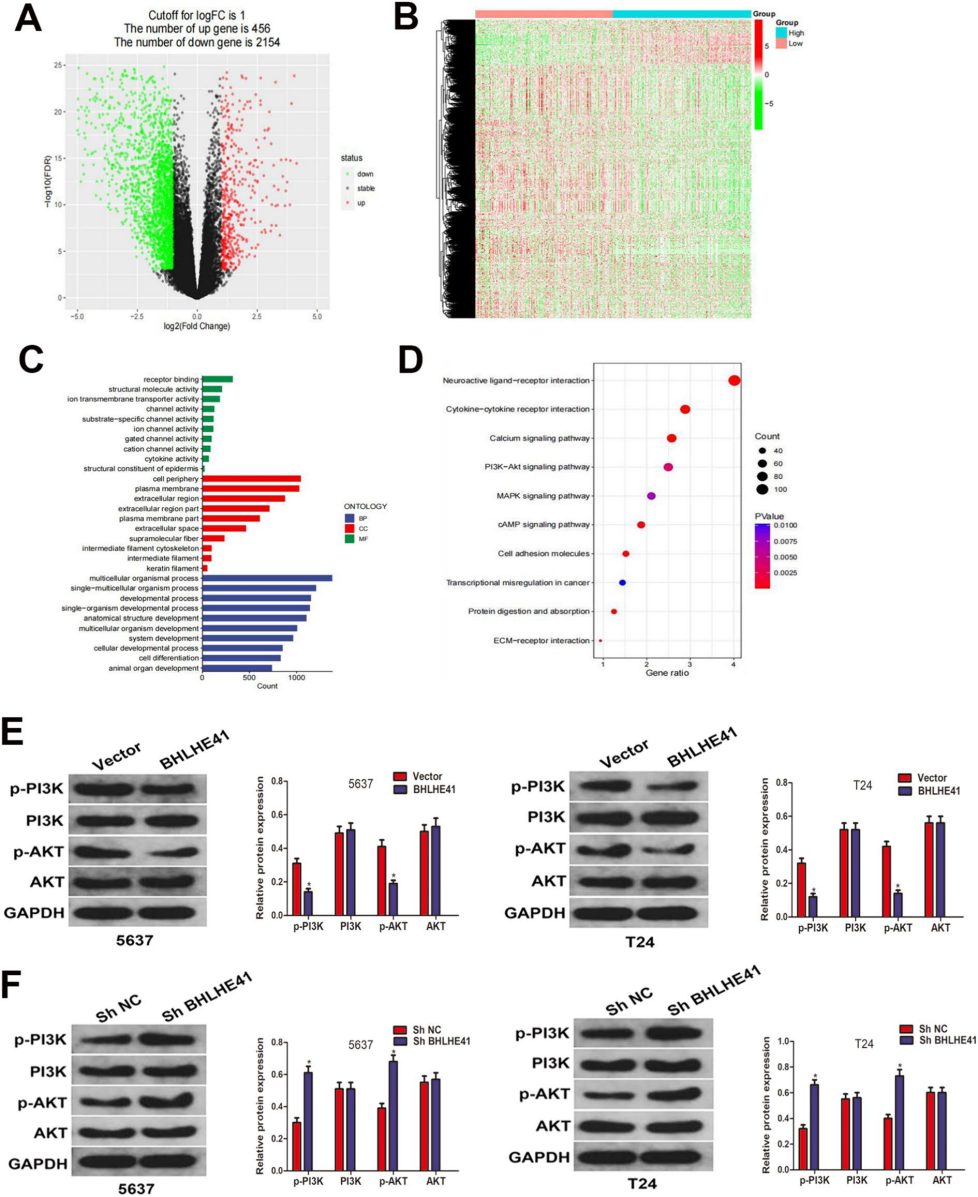
BHLHE41 negatively regulated PI3K/AKT signaling pathway in BCa cells. **A** Volcano plot showing the differentially expressed genes between BHLHE41^high^ and BHLHE41.^low^ groups. **B** Heatmap exhibiting the expression profiles of DEGs in BLCA samples. **C** GO enrichment analysis of DEGs. **D** KEGG enrichment analysis revealed that these DEGs were particularly enriched in PI3K/AKT signaling pathway. **E** Western blot analysis of protein levels of PI3K, p-PI3K, AKT, and p-AKT in BCa cells stably infected with BHLHE41-overexpression or control lentivirus, and quantitative analysis. **F** Western blot analysis was performed to evaluate the effect of BHLHE41 knockdown on the protein levels of PI3K, p-PI3K, AKT, and p-AKT in BCa cells. *P < 0.05

We separated the expression data matrix in the TCGA-BLCA dataset into BHLHE41^high^ and BHLHE41^low^ groups based on the median expression level of BHLHE41 and then performed bioinformatic analysis to evaluate the potential mechanisms of tumor inhibition mediated by BHLHE41. With the criterion of | log2(fold change) |> 1 and FDR < 0.05, a total of 2610 differentially expressed genes (DEGs) were discovered across the two groups (Fig. 4A). Figure 4B shows the expression patterns of these DEGs in BCa samples. These BHLHE41-related DEGs were then subjected to GO enrichment analysis. In terms of biological process, these DEGs were mainly enriched in multicellular organismal process, single-multicellular organism process, and developmental process. With respect to cellular component, cell periphery, plasma membrane, and extracellular region were the three most enriched terms. As for the molecular function, These BHLHE41-related DEGs were particularly enriched in receptor binding, structural molecule activity, and ion transmembrane transporter activity (Fig. 4C). In the KEGG enrichment analysis, we found that these BHLHE41-related DEGs were notably enriched in several signaling pathways, including neuroactive ligand-receptor interaction, cytokine-cytokine receptor interaction, calcium signaling pathway, PI3K-Akt signaling pathway, and MAPK signaling pathway (Fig. 4D). We became particularly interested in the PI3K/AKT signaling pathway, which is a crucial route during carcinogenesis and tumor growth, among the aforementioned enriched pathways in the KEGG study. As a result, we investigated whether BHLHE41 regulated the PI3K/AKT signaling pathway by measuring the protein levels of PI3K, p-PI3K, AKT, and p-AKT. Western blot analysis showed that overexpression of BHLHE41 resulted in a substantial decrease in phosphorylated PI3K and phosphorylated AKT, whereas total protein levels of PI3K and AKT remained steady (Fig. 4E). Conversely, compared to shNeg group, the key PI3K/AKT pathway components such as p-PI3K and p-AKT were increased in the BHLHE41 knockdown group (Fig. 4F). Thus, these results indicated that BHLHE41 negatively regulated PI3K/AKT signaling pathway in BCa cells.

### BHLHE41 interacted with PYCR1 and regulated its stability

**Fig. 5:**
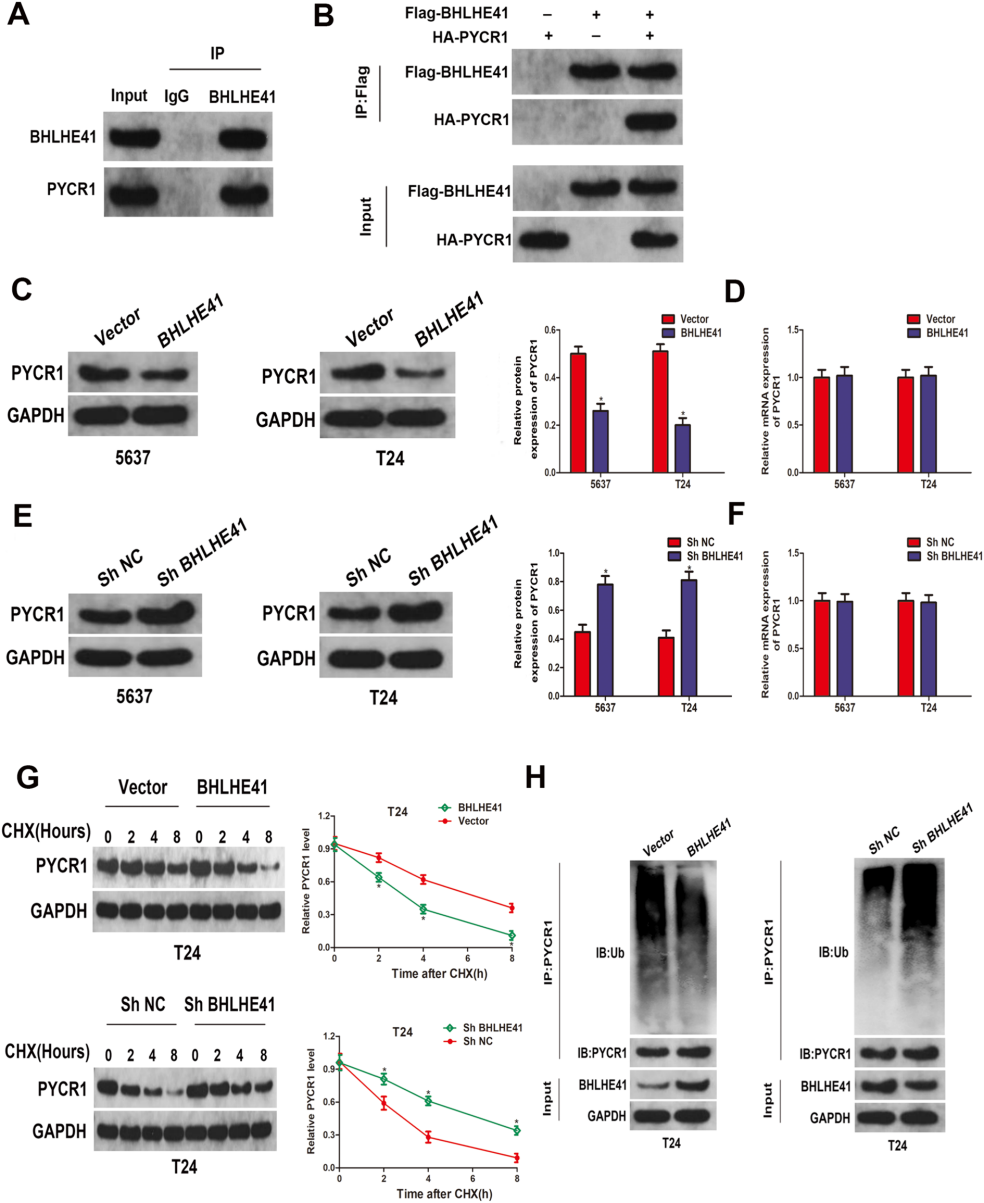
BHLHE41 interacted with PYCR1 and regulated its stability. **A** Immunoprecipitation with anti-BHLHE41 followed by immunoblotting with anti-PYCR1 in T24 cells. **B** Immunoprecipitation with anti-Flag. Cells were transfected with Flag-BHLHE41, HA-PYCR1 or their combination before performing Co-IP assay. **C** Western blot analysis of the effect of BHLHE41 overexpression on PYCR1 protein levels in BCa cells. **D** qRT-PCR analysis was performed to detect the mRNA level of PYCR1 after overexpressing BHLHE41. **E** Western blot analysis of the effect of BHLHE41 knockdown on PYCR1 protein levels in BCa cells. **F** qRT-PCR analysis was performed to detect the mRNA level of PYCR1 after knockdown of BHLHE41. **G** Western blot analysis of PYCR1 protein stability after overexpression or knockdown of BHLHE41. **H** In *vivo* ubiquitination assay was performed to assess the ubiquitination of PYCR1 after overexpression or knockdown of BHLHE41 in T24 cells. *P < 0.05

We tried to identify the potential target of BHLHE41 using the BioGRID portal. Pyrroline-5-carboxylate reductase 1 (PYCR1), a well-documented tumor promoting factor, attracted our attention since it was reported to be dysregulated in bladder cancer and might be participated in regulating PI3K/AKT pathway [25]. We then performed co-immunoprecipitation analysis using the anti-BHLHE41 antibody and anti-PYCR1 antibody in T24 cell lysate, individually. Reciprocally, western blot assay confirmed that endogenous BHLHE41 co-immunoprecipitated with endogenous PYCR1 in the co-immunoprecipitation experiment (Fig. 5A), indicating the endogenous interactions between the two proteins. Besides, we co-transfected the 293 T cells with Flag-BHLHE41 and HA-PYCR1, and the interaction between Flag- BHLHE41 and HA-PYCR1 was also detected (Fig. 5B). To investigate whether BHLHE41 regulated the stability of PYCR1, we examined the expression of PYCR1 in T24 cells with stable BHLHE41 overexpression and knockdown. Surprisingly, it turned out that the protein level of PYCR1 was markedly decreased when BHLHE41 was overexpressed (Fig. 5C), while the protein level of PYCR1 increased when BHLHE41 was knocked down (Fig. 5E). Furthermore, no alternations of PYCR1 mRNA levels were detected with BHLHE41 overexpression or knockdown, according to our qRT-PCR data (Fig. 5D–F). Therefore, we could conclude that BHLHE41 may regulate PYCR1 stability via posttranscriptional modification. Subsequently, the cycloheximide analysis was performed to explore the effect of BHLHE41 on the half-life of PYCR1. As shown in Fig. 5G, western blot assay showed that overexpression of BHLHE41 reduced the half-life of PYCR1, while BHLHE41 knockdown prolonged the half-life of PYCR1. More importantly, in vivo ubiquitination assays revealed that overexpression of BHLHE41 markedly increased the polyubiquitination of PYCR1 in T24 cells, while the level of ubiquitinated PYCR1 was profoundly decreased when BHLHE41 was knocked down (Fig. 5H). Taken together, our results demonstrated that BHLHE41 regulated PYCR1 protein stability through ubiquitin-dependent proteasome degradation manner.

### *BHLHE41 inhibited bladder cancer progression *via* PYCR1-mediated inactivation of PI3K/AKT signaling pathway*

**Fig. 6:**
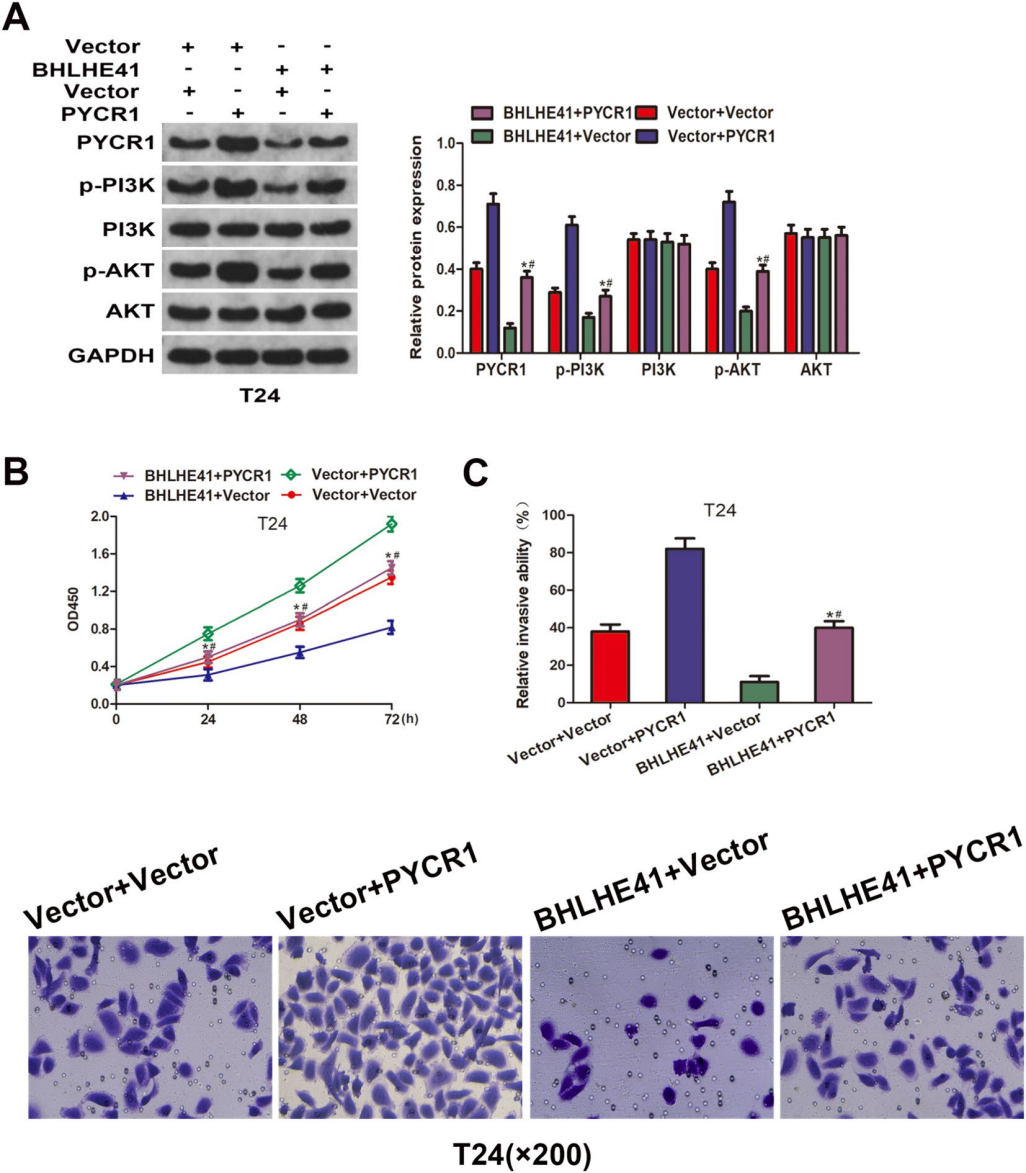
BHLHE41 inhibited bladder cancer progression via PYCR1-mediated inactivation of PI3K/AKT signaling pathway. **A** Western blot analysis of PYCR1, PI3K, p-PI3K, AKT, and p-AKT protein levels in T24 cells stably overexpressing BHLHE41 and transfected with PYCR1 overexpression plasmid. **B**-**C** Cell proliferation and invasion abilities were assessed in T24 cells stably overexpressing BHLHE41 and transfected with PYCR1 overexpression plasmid. *P < 0.05

To clarify the specific role of PYCR1 and BHLHE41 in BCa and further determine whether BHLHE41-mediated inhibition of PI3K/AKT signaling pathway requires PYCR1 degradation, we manipulated the expression of PYCR1 in T24 cells stably overexpressing BHLHE41, and further examined the protein levels of key PI3K/AKT pathway components by western blot assay. As shown in Fig. 6A, overexpressing PYCR1 restored the level of p-PI3K and p-AKT in LV-BHLHE41 cells, indicating that BHLHE41 regulated PI3K/AKT signaling pathway at least partly via PYCR1. Next, we explored the effect of PYCR1 overexpression on cell proliferation, migration, and invasion in T24 cells stably overexpressing BHLHE41. CCK-8 assay revealed that overexpression of PYCR1 significantly restored the proliferation capacity of BHLHE41-overexpressed cells (Fig. 6B). Similarly, the results of transwell invasion assays showed that overexpressing PYCR1 markedly attenuated BHLHE41 overexpression-induced inhibition of cell invasion (Fig. 6C). Altogether, these results suggested that BHLHE41 inhibited bladder cancer progression via PYCR1/PI3K/AKT signaling pathway.

### *BHLHE41 inhibited bladder cancer growth *in vivo

**Fig. 7:**
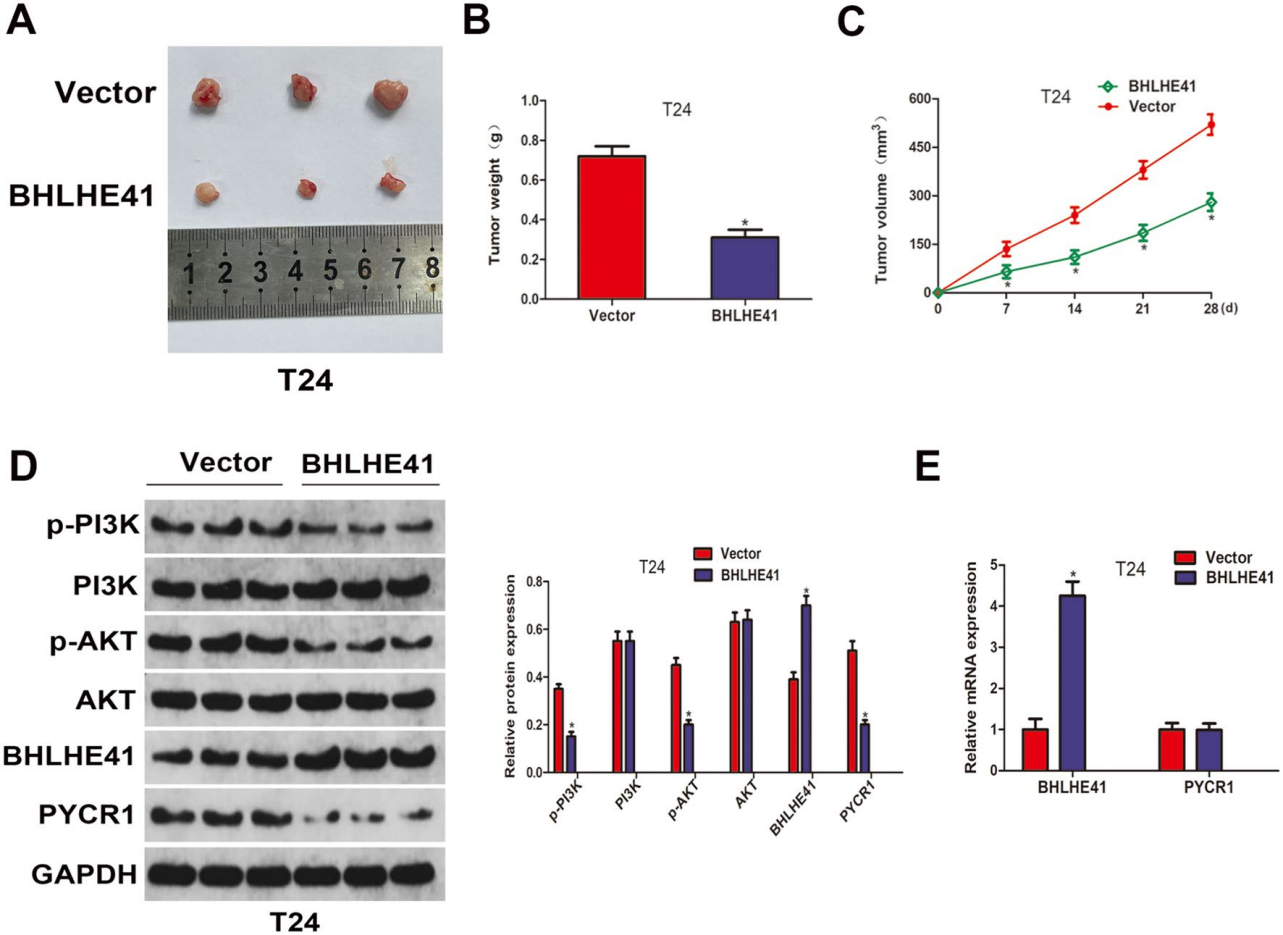
BHLHE41 inhibited bladder cancer growth in vivo. **A** Representative image of xenografts in Vector and BHLHE41 groups. **B** Tumor weights were measured and compared in Vector and BHLHE41 groups. **C** Tumor volumes were monitored every week. **D** Western blot analysis of BHLHE41, PYCR1, PI3K, p-PI3K, AKT, and p-AKT protein levels in xenografts of Vector and BHLHE41 groups, and quantitative analysis. **E** The mRNA levels of BHLHE41 and PYCR1 in xenografts were detected by qRT-PCR assay. *P < 0.05

Finally, to evaluate the anti-tumor effect of BHLHE41 overexpressing in vivo, equal amounts of LV-BHLHE41 and LV-Ctrl T24 cells were subcutaneously injected into the BALB/c nude mice. Tumor volumes were monitored every five days until the mice were sacrificed, and the tumors were resected, photographed and weighted. The overexpression of BHLHE41 in LV-BHLHE41 group was demonstrated by western blot, and was exhibited in Fig. 7D–E. As shown in Fig. 7A–C, the tumor volumes and weights in LV-BHLHE41 group were markedly reduced than those in LV-Ctrl group. Furthermore, the expression levels of key PI3K/AKT pathway components were detected by western blot assay. As shown in Fig. 7D, overexpression of BHLHE41 was found to induced decreased protein levels of PYCR1, p-PI3K, and p-AKT, while the mRNA level of PYCR1 exhibited no difference between LV-Ctrl and LV-BHLHE41 groups (Fig. 7E). Collectively, these findings further supported that overexpression of BHLHE41 inhibited bladder cancer growth in *vivo*, which might be associated with the downregulation of PYCR1 and the inactivation of PI3K/AKT signaling pathway.

## Discussion

Ubiquitination is one of the most common posttranslational modifications, which mediated the intracellular proteins’ turnover and homeostasis. It should be noted that protein ubiquitination is a reversible and dynamic process, precisely determined by several E3 ubiquitin ligases and deubiquitinases [26]. Dysregulated ubiquitination process led to aberrant expression of downstream substrates, which was associated with neurological disorders, cardiovascular diseases, metabolic disorders, and tumorigenesis [27–29]. Previous studies had already revealed the abnormal ubiquitination mechanisms of multiple onco-proteins or tumor suppressors during the development and progression of malignant tumors [30–32]. In bladder cancer, a variety of proteins were aberrantly expressed in tumors and functioned as E3 ubiquitin ligases, involved in the regulation of various biological processes including proliferation, invasion, cell cycle, apoptosis, stemness and chemoresistance [33–36]. For instance, the expression of TRIM38 was shown to be low in bladder cancer, and it was inversely associated with overall survival and advanced clinical features. Overexpression of TRIM38 inhibits the growth of bladder cancer via enhancing the ubiquitinoylation and degradation of GLUT1 [37]. In a separate study, Wang et al. [38] discovered that the E3 ubiquitin ligase RNF126 facilitated the advancement of bladder cancer by boosting the degradation of PTEN, hence activating the EGFR/PI3K/AKT signaling pathway. These investigations suggested that the dysregulated process of ubiquitination affected several intercellular signaling networks or downstream substrates to regulate tumor progression.

In this study, we first revealed that BHLHE41 functions as a tumor suppressor, effectively impeding the progression of bladder cancer. Our investigation revealed that the expression of BHLHE41 was significantly downregulated in both bladder cancer tissues and cell lines. Moreover, there was a clear positive association between the expression levels of BHLHE41 and the overall survival of patients. The role of BHLHE41 in the diagnosis of BCa is still unclear, we believed that using artificial intelligence strategy to analyze the datasets in public resources would provide a clue [39]. Furthermore, our study revealed that the overexpression or suppression of BHLHE41 may effectively hinder or enhance the proliferation, migration, and invasion of bladder cancer cells. This suggests that BHLHE41 plays a crucial role in suppressing the development of bladder cancer. In order to underearth the potential downstream pathway of BHLHE41, we conducted an integrative analysis to identify BHLHE41-related differentially expressed genes based on date from public resources, and then subjected them to functional enrichment analysis. The PI3K/AKT signal pathway, one of the most enriched pathways in the KEGG analysis, had caught our interest due to its well-established involvement in the occurrence and progression of bladder cancer. It was reported that approximately 40% of bladder cancer cases have frequent alterations in the PI3K/AKT signaling. Several potential targets within this pathway have been used for pharmacologic treatments [40, 41]. Thus, we investigate the impact of BHLHE41 on the PI3K/AKT signaling pathway by assessing the phosphorylation of its main components (PI3K and AKT). Our findings demonstrated that upregulation or downregulation of BHLHE41 resulted in a reduction or rise in the phosphorylation level of PI3K and AKT, suggesting that BHLHE41 has a negative regulatory effect on the PI3K/AKT signaling pathway in bladder cancer.

PYCR1 belongs to the PYCR family that comprises three isozymes PYCR1, PYCR2, and PYCRL [42]. PYCR1 is widely expressed in human tissues and it was mainly responsible for the synthesis of proline [43]. Previous studies reported that PYCR1 exerted a significant role in the malignant progression of multiple cancers. Gao et al. [44] found that PYCR1 was highly expressed in lung adenocarcinoma cell lines and tissues, higher PYCR1 expression predicted worst prognosis in LUAD patients. Further studies revealed that knockdown of PYCR1 curbed cell malignant behaviors via affecting the JAK/STAT signaling pathway. In bladder cancer, Du et al. [25] found that PYCR1 was highly expressed in bladder cancer tissues and increased expression of PYCR1 correlated with decreased survival rates. Overexpression of PYCR1 accelerated cellular proliferation and invasion via activating Akt/WNT/β-catenin signaling. To further underearth the possible mechanism by which BHLHE41 regulated the activity of PI3K/AKT signaling, we tried to identify the interacting proteins of BHLHE41. PYCR1, a well-characterized tumor promoting factor, attracted our attention for the following reasons: (1) PYCR1 was frequently dysregulated in malignant tumors; (2) PYCR1 might be interact with BHLHE41; (3) PYCR1 might be participated in regulating PI3K/AKT signaling. As expected, Co-IP assay demonstrated the interaction of BHLHE41 and PYCR1. Furthermore, BHLHE41 negatively regulated the protein level of PYCR1, while had no effect on its mRNA level, indicating that BHLHE41 regulated the expression of PYCR1 through a post-transcription mechanism. BHLHE41 was previously reported to promoted the proteasomal degradation of hypoxia-inducible factors (HIF-1α and HIF-1β) by direct binding and presenting them to the proteasome [45]. Thus, we speculated that BHLHE41 might regulate the degradation of PYCR1. To address this question, we explore the effect of BHLHE41 on the stability of PYCR1 through cycloheximide assay. The results showed that overexpression of BHLHE41 decreased the protein level of PYCR1 at the indicated time points after the treatment of cycloheximide, while knockdown of BHLHE41 had the opposite effect, indicating that BHLHE41 decreased the stability of PYCR1. Furthermore, in *vivo* ubiquitination assays revealed that BHLHE41 promoted the polyubiquitination of PYCR1. Collectively, our results demonstrated that BHLHE41 regulated PYCR1 protein stability through ubiquitin-dependent proteasome degradation manner. Furthermore, rescue experiments showed that PYCR1 overexpression restored the level of p-PI3K and p-AKT in BHLHE41-overexpressed cells, Meanwhile, overexpressing PYCR1 markedly attenuated BHLHE41 overexpression-induced inhibition of cell proliferation and invasion. In all, we found for the first time that BHLHE41 acted as a novel E3 ligase for PYCR1 ubiquitination degradation in the current study. BHLHE41 played a suppressive role in the progression of bladder cancer via regulation of PYCR1 stability and thus inactivation of PI3K/AKT signaling pathway.

There were some shortcomings in our study. First, the E3 ligase activity of BHLHE41 should also be detected in *vitro*. Second, the effect of BHLHE41 on the apoptosis, stemness, and chemoresistance warranted further examined. Besides, the ubiquitination site of PYCR1 by BHLHE41 needed to be explored.

In summary, we examined the expression level and the role of BHLHE41 in bladder cancer, and identified that BHLHE41 inhibited bladder cancer progression via regulation of PYCR1 stability and thus inactivating of PI3K/AKT signaling pathway. Our study strongly implied that BHLHE41 might be a useful prognostic biomarker and might be a potential therapeutic target for bladder cancer intervention.

## Data Availability

No datasets were generated or analysed during the current study.
